# Supplementary material for: DNA oligonucleotides encoding suppressor tRNA induce readthrough of premature termination codon
Source: Mol Ther Nucleic Acids. 2026 Sep 24;37(4):103110. doi: 10.1016/j.omtn.2026.103110 (PMC13670950; doi:10.1016/j.omtn.2026.103110)
Supplement: Supplementary file 1 [file mmc1.pdf]

OMTN, Volume *37*

Supplemental information

DNA oligonucleotides encoding suppressor tRNA induce readthrough of premature termination codon

Haruka Yamamoto, Peixin Zhu, and Keisuke Yonehara

Table S1. Sequences of DNA and RNA oligonucleotides

Name	Sequence (5'-3')	
tRT6-sense	GGCTCTGTGGCGCAATGGATAGCGCATTGGACTTCAAATTCAAAGGTTGCGGGTTCGAGTCCCGTCAGAGTCGCCA	DNA
tRT6-antisense	TGGCGACTCTGACGGGACTCGAACCCGCAACCTTTGAATTTGAAGTCCAATGCGCTATCCATTGCGCCACAGAGCC	DNA
tRT6-mut	GGCTCTGTGGCGCAATGGATAGCGCATTGGACTTCGAATTCAAAGGTTGCGGGTTCGAGTCCCGTCAGAGTCGCCA	DNA
suptRNA-Quaa	GGCCCCATGGTGTAATGGTtAGCACTCTGGACTTTAAATCCAGCGATCCGAGTTCAAATCTCGGTGGGACCTCCA	DNA
suptRNA-Yuag	CCTTCGATAGCTCAGTTGGTAGAGCGGAGGACTCTAGATCCTTAGGTCGCTGGTTCGATTCCGGCTCGAAGGACCA	DNA
Oligo 1	GGCTCTGTGGCGCAATGGATAGCGCATTGGACTTCAAA	DNA
Oligo 2	AAGTCCAATGCGCTATCCATTGCGCCACAGAGCC	DNA
Oligo 3	TTCAAAGGTTGCGGGTTCGAGTCCCGTCAGAGTCGCCA	DNA
Oligo 4	TGGCGACTCTGACGGGACTCGAACCCGCAACCTTTGAATTTG	DNA
tRT6-RNA	GGCUCUGUGGCGCAAUGGAUAGCGCAUUGGACUUCAAAUUCAAAGGUUGCGGGUUCGAGUCCCGUCAGAGUCGCCA	RNA
tRT6-mut-RNA	GGCUCUGUGGCGCAAUGGAUAGCGCAUUGGACUUCGAAUUCAAAGGUUGCGGGUUCGAGUCCCGUCAGAGUCGCCA	RNA
tRT6 miRNA	CGAACCCGCAACCUUUGAAUUUGA	RNA
Control miRNA	UUGAACUCGGAUCGCUGGAUUUAA	RNA
tRT6-Cy5	CCCGCAACCTTTGAATTTGA	DNA
human-U6-snRNA-DY782	TGGAACGCTTCACGAATTTG	DNA
tRT6-qPCR-F	CCCGCAACCTTTGAATTTGA	DNA
tRNA-Ala-AGC-2-1-qPCR-F	TAGCTCAGTGGTAGAGCGCG	DNA
tRNA-qPCR-common-R	CTAAGAGCAAGAAGAAGCCT	DNA
