# Supplementary material for: DNA oligonucleotides encoding suppressor tRNA induce readthrough of premature termination codon
Source: Mol Ther Nucleic Acids. 2026 Sep 24;37(4):103110. doi: 10.1016/j.omtn.2026.103110 (PMC13670950; doi:10.1016/j.omtn.2026.103110)
Supplement: Supplementary file 2 [file mmc2.pdf]

Molecular Therapy
Nucleic Acids
Original Article

DNA oligonucleotides encoding suppressor tRNA induce readthrough of premature termination codon

Haruka Yamamoto,[1] Peixin Zhu,[1] and Keisuke Yonehara[1,2,3]

[1]Department of Gene Function and Phenomics, National Institute of Genetics, Mishima 411-8540, Japan; [2]Genetics Program, Graduate Institute for Advanced Studies, SOKENDAI, Hayama 240-0115, Japan; [3]Department of Systems Physiology, Yokohama City University Graduate School of Medicine, Yokohama 236-0004, Japan

Nonsense mutation is a type of DNA mutation that converts sense codon in mRNA into premature termination codon (PTC), causing protein truncation. Recent studies have revealed that suppressor tRNAs, which read the stop codon instead of the natural codon and encode the desired amino acid, induce PTC readthrough and restore protein function. However, RNA is prone to degradation, presenting numerous challenges for tissue delivery in gene therapy. Here, we show that DNA oligonucleotides encoding suppressor tRNA sequences but without additional Pol III promoters induce PTC readthrough in cultured cells. Sequence-matched microRNAs specifically inhibited PTC readthrough mediated by the transfected DNA oligonucleotides. Furthermore, treatment with an RNA Pol III inhibitor significantly reduced DNA oligonucleotide-mediated PTC readthrough. These findings support the conclusion that functional suppressor tRNAs are transcribed from DNA oligonucleotides in cultured cells. These results suggest that DNA oligonucleotides hold potential as a novel therapeutic approach enabling suppressor tRNA expression for nonsense mutation repair.

INTRODUCTION

Nonsense mutations are DNA alterations that convert a sense codon into a premature termination codon (PTC) within coding regions, causing the ribosome to terminate protein synthesis prematurely and produce truncated proteins. These truncated proteins often lack essential functional domains, rendering them nonfunctional or severely impaired. Nonsense mutations are an important class of pathogenic variants in humans and are estimated to account for approximately 11% of known genetic diseases.[1] To restore the function of truncated proteins induced by PTCs, several chemical approaches, such as aminoglycoside antibiotics,[2] PTC124,[3] and NVS1.1,[4] have been developed to promote PTC readthrough and allow amino acid incorporation, thereby producing full-length proteins. However, these chemical approaches generally do not ensure the insertion of a specific amino acid at the PTC site, which is required for complete restoration of protein function. Consequently, even when full-length proteins are produced, their biological activity may not be fully recovered and these compounds, such as aminoglycoside antibiotics, may pose a risk of severe ototoxicity and nephrotoxicity, as previously reviewed.[5]

Anticodon-engineered tRNAs, known as suppressor tRNAs, have emerged as an alternative strategy to restore protein synthesis at PTC sites.[6] Suppressor tRNAs specifically recognize premature stop codons through their engineered anticodon sequences and insert a predefined amino acid in place of the PTC.[7] In contrast to chemical readthrough agents, suppressor tRNAs enable selective targeting of specific stop codons and the incorporation of the correct amino acid at the mutation site, providing a more precise and reliable approach for restoring the native function of the affected protein. Recently, several *in vivo* studies have reported the use of AAV vectors or lipid nanoparticle for the delivery of suppressor tRNAs.[8,9] Although these delivery platforms have enabled substantial advances in suppressor tRNA therapeutics, each has inherent limitations. AAV vectors are constrained by limited cargo capacity, may elicit immune responses, and can result in prolonged transgene expression,[10] whereas lipid nanoparticles often exhibit tissue-dependent delivery efficiency and inefficient intracellular delivery.[11] These limitations highlight the need for alternative approaches for suppressor tRNA delivery. Here, we sought to develop a transient, vector-free strategy for suppressor tRNA delivery and demonstrate that synthetic DNA oligonucleotides encoding suppressor tRNAs are capable of inducing PTC readthrough in cultured cells.

RESULTS

DNA oligonucleotides induce PTC readthrough

To evaluate PTC readthrough via suppressor tRNAs in cultured cells, we employed the tRT6 suppressor tRNA, which incorporates arginine (Arg, R) at the UGA stop codon,[9] To confirm the activity of tRT6, we designed a variant, tRT6-mut, in which the anticodon sequence was changed to UCG, allowing recognition of the CGA

Received 6 February 2026; accepted 21 September 2026;
https://doi.org/10.1016/j.omtn.2026.103110.

Correspondence: Keisuke Yonehara, Department of Gene Function and Phenomics, National Institute of Genetics, Mishima 411-8540, Japan.
E-mail: yonehara.kei.vq@yokohama-cu.ac.jp

© 2026 The Author(s).
Published by Elsevier Inc. on behalf of The American Society of Gene and Cell Therapy.
This is an open access article under the CC BY license (http://creativecommons.org/licenses/by/4.0/).

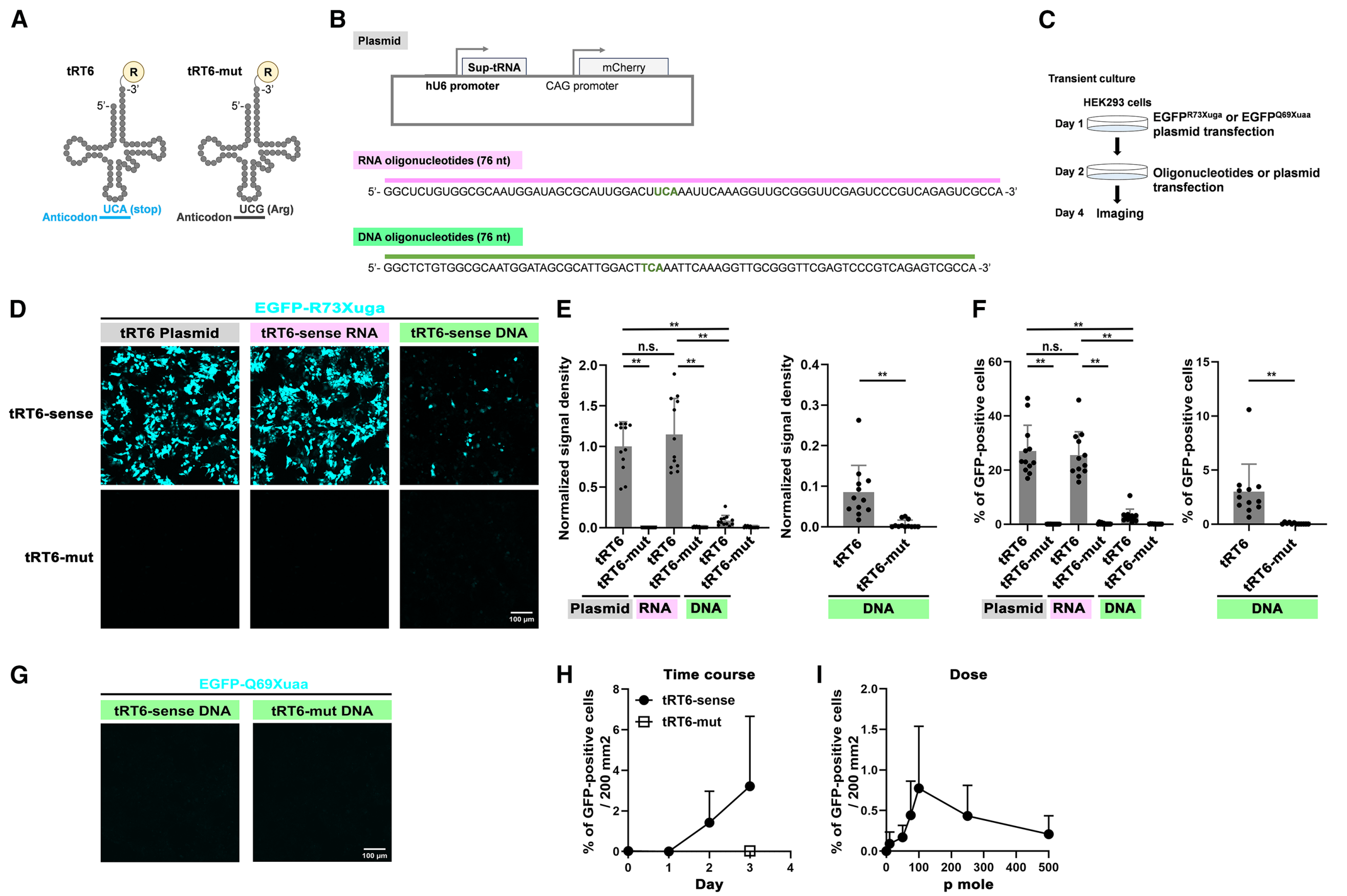

Figure 1. Functional assay of suppressor tRNAs for EGFP nonsense mutants

(A) Diagrams of the tRT6 suppressor tRNA recognizing the UGA stop codon and the tRT6-mut variant recognizing the CGC arginine codon. (B) Schematic of suppressor tRNA cassettes delivered as plasmids, synthetic RNA oligonucleotides, or synthetic DNA oligonucleotides. (C) Experimental workflow of the PTC readthrough assay using EGFP nonsense mutant reporters. (D) Representative fluorescence images of cells expressing the $EGFP^{R73Xuga}$ nonsense reporter and transfected with the indicated tRNA cassettes. (E) Left, quantification of EGFP-positive cell density corresponding to (D). Fluorescence densities were normalized to DAPI signals to account for differences in total cell number across images. $n = 12$ images from four independent experiments. One-way ANOVA with post-hoc Tukey test ($**p < 0.01$, n.s., not significant). Right, rescaled plotting of the DNA oligonucleotide samples. Student's t test ($**p < 0.01$). (F) Left, the fraction of EGFP-positive cell corresponding to (D). $n = 12$ images from four independent experiments. One-way ANOVA with post-hoc Tukey test ($**p < 0.01$, n.s., not significant). Right, rescaled plotting of the DNA oligonucleotide samples. Student's t test ($**p < 0.01$). (G) Representative fluorescence images of cells expressing the $EGFP^{Q69Xuaa}$ nonsense reporter transfected with tRT6 DNA oligonucleotides or tRT6-mut DNA oligonucleotides. (H) Quantification of the fraction of GFP-positive cells relative to DAPI-positive nuclei. $n = 5$–8 images per time point from three independent experiments. (I) Quantification of the fraction of GFP-positive cells relative to DAPI-positive nuclei in cells transfected with the indicated amounts of the tRT6-sense DNA oligonucleotide. $n = 7$–8 images per dose from three independent experiments. Scale bars indicate 100 μm.

arginine codon instead of the target stop codon (Figure 1A). Its activity was then assessed using the EGFP nonsense mutant reporter in a PTC readthrough assay. To evaluate the ability of various molecules to induce PTC readthrough, we transfected cells with various types of molecules encoding suppressor tRNAs using expression plasmids, synthetic RNA oligonucleotides, or synthetic DNA oligonucleotides (Figure 1B). Prior to transfection with these molecules, cells were pre-transfected with the $EGFP^{R73Xuga}$ reporter plasmid one day earlier. This plasmid contains the UGA stop codon as a PTC at amino acid position 73 of EGFP (Figure 1C). We observed strong GFP fluorescence in cells transfected with the tRT6 plasmid (Figure 1D), consistent with the previous study.[9] In contrast, cells transfected with tRT6-mut, which does not recognize the stop codon due to the anticodon mismatch, showed no detectable GFP signal (Figure 1D). We next found that tRT6 RNA oligonucleotide elicited relatively strong GFP fluorescence within cells, whereas the tRT6-mut RNA oligonucleotide did not (Figure 1D). Interestingly, cells transfected with the DNA-sense oligonucleotide encoding tRT6 showed significant PTC readthrough compared to cells transfected with the oligonucleotide encoding tRT6-mut. The readthrough efficiency in samples transfected with plasmid and RNA was similar, but in samples transfected with DNA, it exhibited approximately 10% of the readthrough observed under plasmid or RNA conditions (Figure 1E). A similar trend was observed when the data were

analyzed as the fraction of GFP-positive cells relative to DAPI-positive nuclei (Figure 1F). Next, we verified the target sequence specificity of DNA oligonucleotides encoding suppressor tRNAs. No detectable PTC readthrough was observed in another EGFP nonsense mutant reporter, Q69X that contains the UAA stop codon at amino acid position 69 (Figure 1G). To further characterize the activity of DNA oligonucleotide-derived suppressor tRNAs, we performed time-course and dose-response analyses. In the time-course experiment, the fraction of GFP-positive cells increased progressively over time following transfection with the tRT6 DNA oligonucleotide, whereas no GFP-positive cells were detected in cells transfected with the tRT6-mut DNA oligonucleotide at day 3 (Figure 1H). We next examined the effect of DNA oligonucleotide dose on PTC readthrough. GFP-positive cells were detected across a range of DNA oligonucleotide doses, with the highest fraction observed at a dose of 100 pmol (Figure 1I). These results indicate that DNA oligonucleotide-derived suppressor tRNA activity increases over time and that the optimal dose depends on the experimental scale and transfection method. Taken together, these findings demonstrate that the DNA oligonucleotide encoding the suppressor tRNA can induce PTC readthrough in cultured cells, albeit with low efficiency.

Previous studies of suppressor tRNAs have demonstrated PTC readthrough using vector-based expression systems or synthesized RNA oligonucleotides.[8,9,12,13] However, to our knowledge, no study has reported readthrough activity derived from DNA oligonucleotides. Therefore, we focused on DNA oligonucleotide-mediated PTC readthrough in the subsequent analyses of this study. To determine whether suppressor tRNA production depends on DNA strand orientation, we compared sense and antisense oligonucleotides individually or in combination (Figures 2A and 2B). In addition to tRT6-sense oligonucleotides, GFP fluorescence was also observed in cells transfected with tRT6-antisense DNA oligonucleotides compared to non-transfected controls (Figure 2C). Notably, mixtures of sense and antisense oligonucleotides elicited GFP signal levels comparable to those observed when used alone (Figure 2D). A similar trend was observed when the data were analyzed as the fraction of GFP-positive cells relative to DAPI-positive nuclei (Figure 2E). These results suggest that sense or antisense alone, and their combinations, can express functional suppressor tRNAs for PTC readthrough in the $EGFP^{R73Xuga}$ nonsense reporter. We next investigated a more minimal DNA oligonucleotide design in which the double-stranded tRT6 DNA sequence was divided into four split DNA oligonucleotides (oligos 1–4). One partial duplex was formed by oligos 1 and 2, whereas the other was formed by oligos 3 and 4. The two duplexes were designed to be connected through complementary three-nucleotide overhangs between oligos 1 and 4 (Figure 2F). We observed PTC readthrough of $EGFP^{R73Xuga}$ nonsense reporter when all four fragments (oligos 1 + 2+3 + 4) were co-transfected into cells (Figure 2G). In contrast, combinations of the sense short fragments (oligos 1 + 3) or pairs consisting of a sense fragment and its complementary antisense fragment (oligos 1 + 4) did not show detectable GFP signals compared to the four-fragment combination (Figures 2H and 2I). These results indicate that the presence of both complementary strands is required for the expression of functional suppressor tRNA.

Next, we examined whether DNA oligonucleotide-derived suppressor tRNAs could be applied to nonsense mutations containing different stop codons. We generated additional EGFP nonsense reporter constructs carrying UAA or UAG premature termination codons ($EGFP^{R73Xuaa}$ and $EGFP^{R73Xuag}$) and designed the corresponding suppressor tRNAs, sup-tRNA-Quaa, and sup-tRNA-Yuag, to recognize UAA and UAG, respectively. We observed PTC readthrough in cells transfected with DNA oligonucleotides encoding the corresponding suppressor tRNAs, demonstrating that this strategy is applicable to all three stop codons (Figures 3A and 3B). To facilitate quantitative evaluation of PTC readthrough while preserving single-cell resolution, we next developed a dual fluorescence reporter consisting of miRFP670, a UGA PTC, a P2A peptide sequence, and EGFP arranged in tandem (Figure 3C). In this reporter, miRFP670 is constitutively expressed in transfected cells, whereas EGFP fluorescence is detected only following suppressor tRNA-mediated readthrough of the UGA stop codon. We observed PTC readthrough of the dual reporter in miRFP670-expressing cells transfected with the tRT6 plasmid or tRT6-sense DNA oligonucleotide but not with the corresponding tRT6-mut plasmid or DNA oligonucleotide (Figures 3D and 3E). These results were consistent with those obtained using the GFP nonsense reporter assay.

Suppressor tRNAs are expressed from DNA oligonucleotides

Lastly, assuming that the DNA oligonucleotides actually produce suppressor tRNAs, we examined the possibility that their activity could be inhibited by antisense microRNAs (miRNAs) that form base pairs with the anticodon and variable loops of the suppressor tRNA sequences (Figure 4A).[14] To verify this, cells pre-transfected separately with the $EGFP^{R73X}$ expression plasmid and the tRT6-antisense DNA oligonucleotides were then transfected with either sequence-matched miRNAs or sequence-mismatched control miRNAs (Figure 4B). In this experiment, the tRT6-antisense DNA oligonucleotides were used as the source of suppressor tRNAs to avoid the possibility that sequence-matched miRNAs might bind to tRT6-sense DNA oligonucleotides and interfere with their function. Cells transfected with sequence-matched tRT6 miRNAs showed significantly reduced GFP signal intensity compared to untransfected cells and cells transfected with sequence-mismatched control miRNAs (Figures 4C and 4D), implying that functional suppressor tRNAs are generated from DNA oligonucleotides. To directly assess suppressor tRNA expression, we first performed northern blot analysis using a Cy5-labeled probe specific for the tRT6 suppressor tRNA. A distinct tRT6 signal was detected in cells transfected with the tRT6 expression plasmid, confirming the specificity of the probe (Figure 4E). In contrast, no specific tRT6 signal was detected in cells transfected with either tRT6-sense or tRT6-antisense DNA oligonucleotides (Figure 4E). This likely reflects suppressor tRNA expression levels below the detection limit of the assay, consistent with the relatively low PTC readthrough efficiency observed in our system. We next employed a tRNA-adapted quantitative reverse-transcription

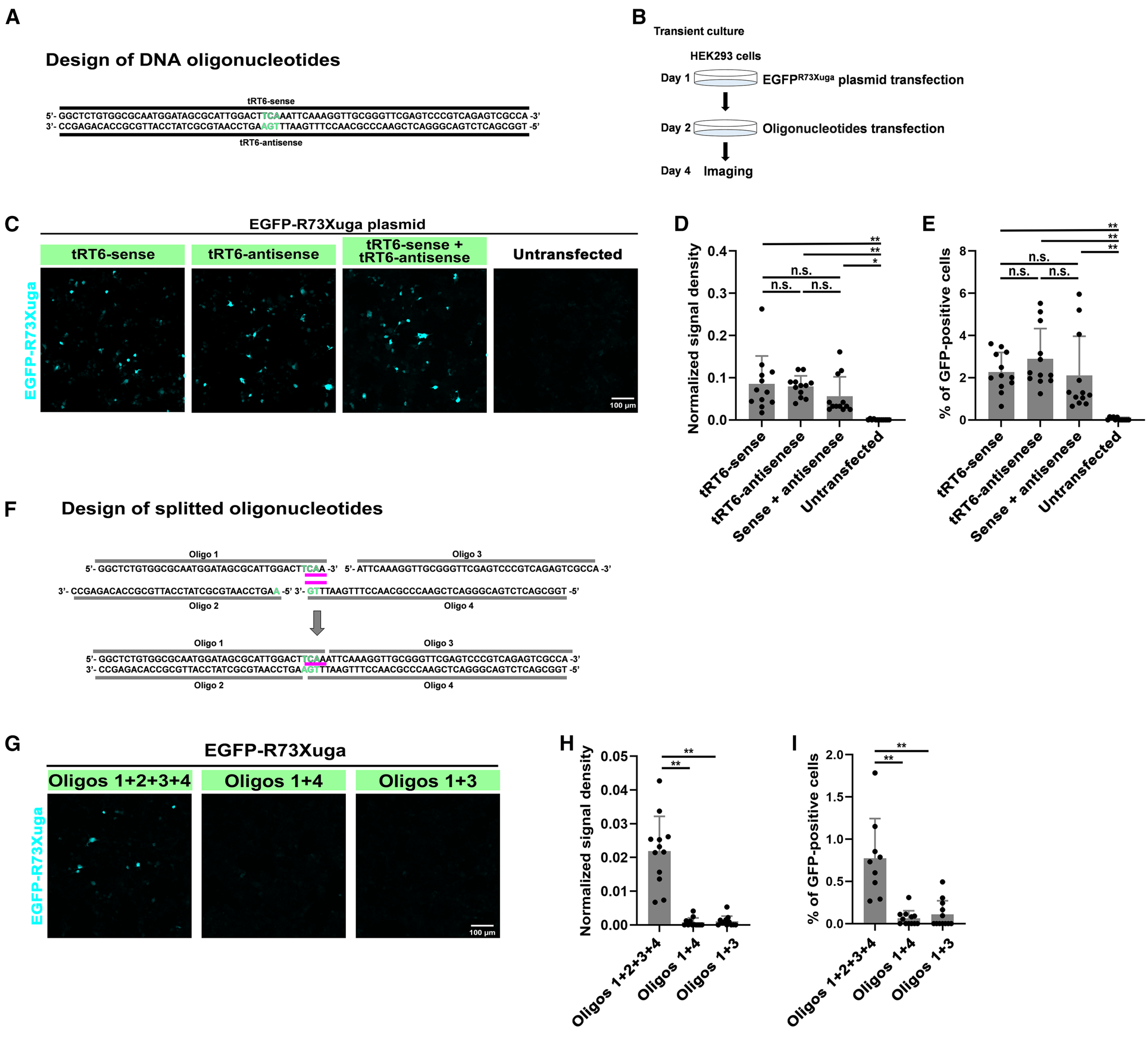

Figure 2. Transient suppressor tRNA expression from DNA oligonucleotides

(A) Schematic of tRT6-sense and tRT6-antisense DNA oligonucleotides. (B) Experimental workflow of the PTC readthrough assay using the EGFP^{R73Xuga} nonsense reporter. (C) Representative fluorescence images of cells expressing the EGFP^{R73X} nonsense reporter, treated with tRT6-sense, tRT6-antisense, tRT6-sense + tRT6-antisense, or untransfected control. (D) Quantification of EGFP-positive cell density corresponding to (C). Fluorescence densities were normalized to DAPI signals to account for differences in total cell number across images. The tRT6-sense data are identical to those in Figure 1E (tRT6-sense DNA), as both experiments were performed simultaneously using the same experimental batches, and is re-plotted here for comparison. $n = 12$ images from four independent experiments. One-way ANOVA with post-hoc Tukey test (**$p < 0.01$, *$p < 0.05$, n.s., not significant). (E) Quantification of the fraction of GFP-positive cells relative to DAPI-positive nuclei corresponding to (C). $n = 12$ images from four independent experiments. One-way ANOVA with post-hoc Tukey test (**$p < 0.01$, n.s., not significant). (F) Schematic of split DNA sequences of tRT6 oligonucleotides. One partial duplex was formed by oligos 1 and 2, whereas the other was formed by oligos 3 and 4. The two duplexes are connected through a three-nucleotide complementary sequence between oligos 1 and 4 (highlighted in magenta). The anticodon region is highlighted in light green. (G) Representative fluorescence images of cells expressing the EGFP^{R73Xuga} nonsense reporter and transfected with the indicated split DNA oligonucleotides. (H) Quantification of EGFP-positive cell density corresponding to (F). Fluorescence densities were normalized to DAPI signals to account for differences in total cell number across images. $n = 12$ images from four independent experiments. One-way ANOVA with post-hoc Tukey's test (**$p < 0.01$, n.s., not significant). (I) Quantification of the fraction of GFP-positive cells relative to DAPI-positive nuclei corresponding to (G). $n = 12$ images from four independent experiments. One-way ANOVA with post-hoc Tukey test (**$p < 0.01$, n.s., not significant). Scale bars indicate 100 μm.

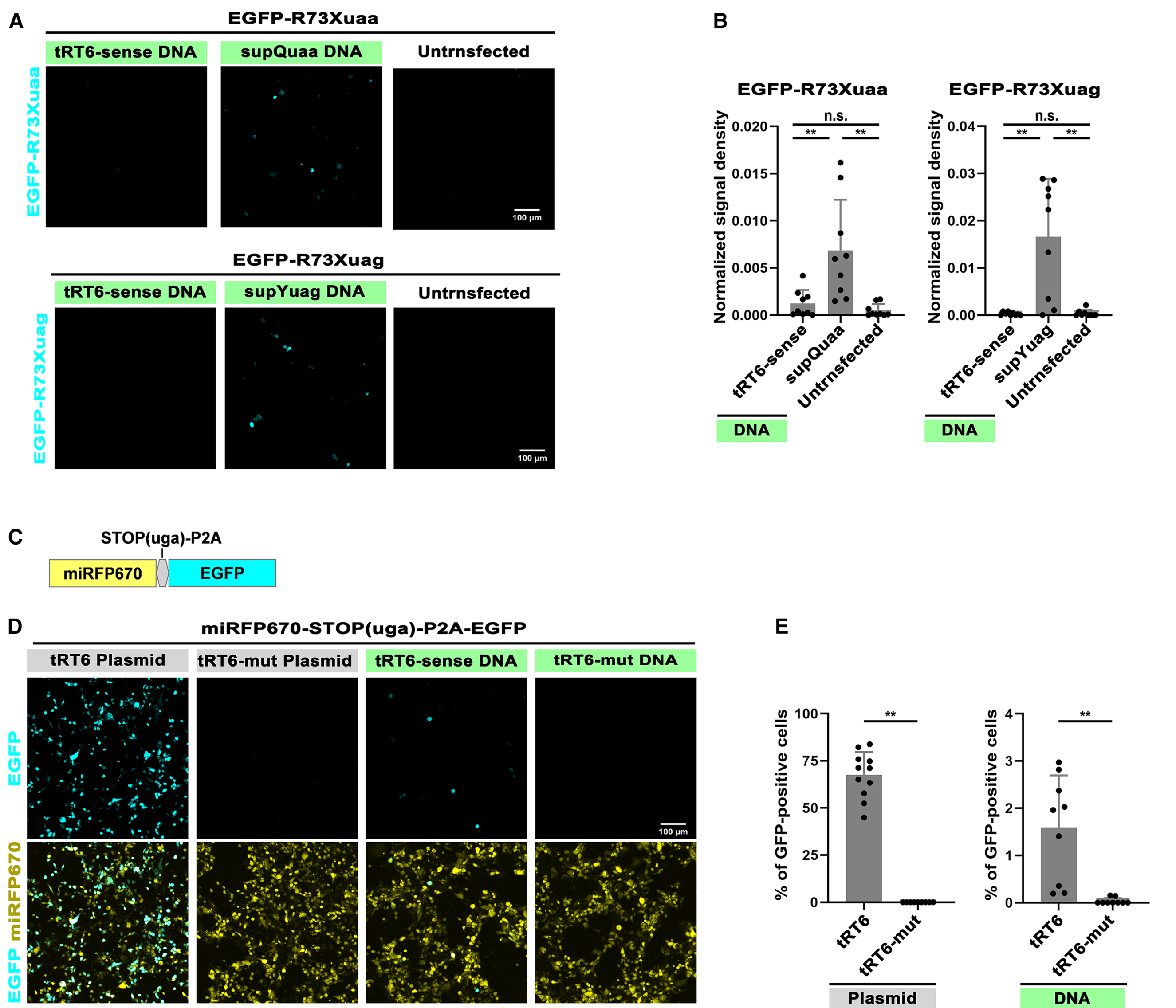

Figure 3. Applicability of DNA oligonucleotide-derived suppressor tRNAs

(A) Representative fluorescence images of cells expressing the $\text{EGFP}^{\text{R73Xuaa}}$ nonsense reporter $\text{EGFP}^{\text{R73Xuag}}$ and transfected with the indicated DNA oligonucleotides. (B) Quantification of GFP-positive cell density corresponding to (A). Fluorescence densities were normalized to DAPI signals to account for differences in total cell number across images. *n* = 9 images from three independent experiments. One-way ANOVA with post-hoc Tukey's test (***p* < 0.01, n.s., not significant). (C) Schematic of the miRFP670-stop-EGFP dual reporter. (D) Representative fluorescence images of cells expressing the miRFP670-stop-EGFP dual reporter and transfected with the indicated plasmids or DNA oligonucleotides. (E) Quantification of the fraction of GFP-positive cells relative to miRFP670-positive cells corresponding to (D). *n* = 9–11 images from three independent experiments. Student's *t* test (***p* < 0.01). Scale bars indicate 100 µm.

PCR (RT-qPCR) approach based on the Nano-tRNAseq library preparation method[15] (Figure 4F). qPCR analysis of the resulting tRNA libraries showed comparable expression of the endogenous reference tRNA, Ala-AGC-2-1, across all samples (Figure 4G). In contrast, lower Ct values for the tRT6 suppressor tRNA were observed in cells transfected with the tRT6 plasmid or tRT6 DNA oligonucleotides than in untransfected controls, indicating successful detection of exogenously expressed suppressor tRNA (Figure 4G). Notably, both the tRT6-sense and tRT6-antisense DNA oligonucleotides increased tRT6 suppressor tRNA expression by more than 100-fold relative to the untransfected control (Figure 4H). These results are consistent with our observation that both the sense and antisense DNA oligonucleotides induced PTC readthrough. To investigate the mechanism underlying suppressor tRNA expression, we next examined the involvement of RNA polymerase III (Pol III) by treating cells with the Pol III inhibitor ML-60218 (Figure 4I). PTC

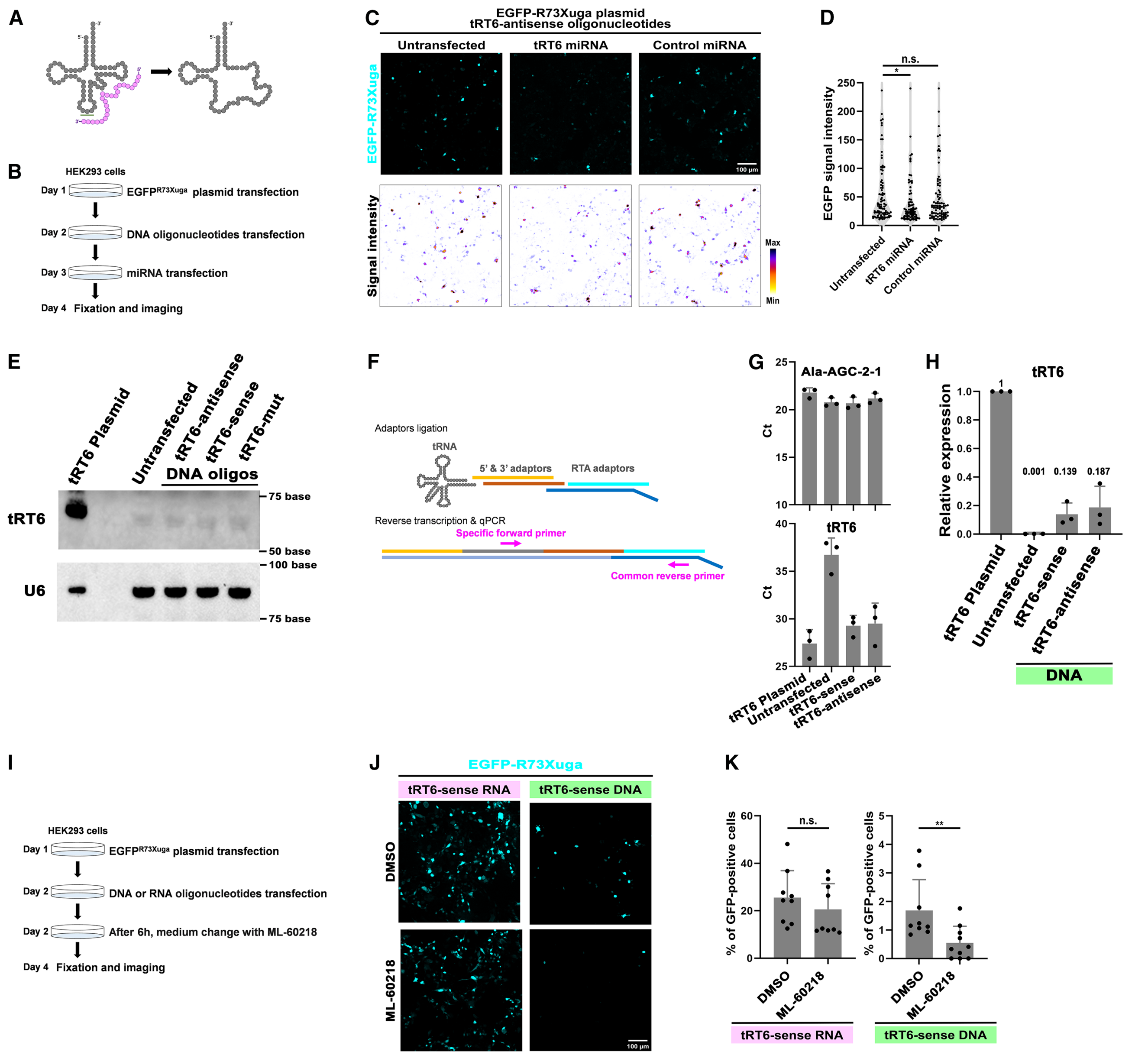

Figure 4. Mechanism underlying suppressor tRNA expression from DNA oligonucleotides

(A) Schematic of suppressor tRNA cassettes delivered as plasmids, synthetic RNA oligonucleotides, or synthetic DNA oligonucleotides. (B) Experimental workflow of the PTC readthrough assay using the $EGFP^{R73Xuga}$ reporter. (C) Representative fluorescence images of cells transfected with the $EGFP^{R73Xuga}$ nonsense reporter and tRT6 antisense oligonucleotides, treated with tRT6 miRNA, control miRNA, or untransfected control. (D) Quantification of $EGFP^{R73Xuga}$ signal intensities of putative transfected cells corresponding to (C). $n = 86$ cells for untransfected control, $n = 81$ cells for tRT6 miRNA, and $n = 86$ cells for control miRNA, from three independent experiments. One-way ANOVA with post-hoc Dunnett's test (* $p < 0.05$, n.s., not significant). (E) Northern blot analysis of the tRT6 suppressor tRNA in small RNAs isolated from untransfected cells and cells transfected with the tRT6 plasmid, tRT6 DNA oligonucleotides. Human U6 served as a loading control. (F) Schematic of tRNA library preparation for qPCR (G) RT-qPCR analysis of Ala-AGC-2-1 (top) and tRT6 suppressor tRNA (bottom). Ct values are shown for each sample. $n = 3$ independent experiments. (H) Relative expression of the tRT6 suppressor tRNA, normalized to the tRT6 plasmid sample. $n = 3$ independent experiments. (I) Experimental workflow of the PTC readthrough assay with ML-60218. (J) Representative fluorescence images of cells expressing the $EGFP^{R73Xuga}$ nonsense reporter, transfected with the tRT6 RNA or DNA oligonucleotide, and treated with ML-60218 or DMSO. (K) Quantification of the fraction of GFP-positive cells relative to DAPI-positive nuclei in cells transfected with the tRT6 RNA oligonucleotide (left) or the tRT6 DNA oligonucleotide (right). $n = 9$ images from three independent experiments. Student's t test (** $p < 0.01$; n.s., not significant). Scale bars indicate 100 μm.

readthrough mediated by synthetic RNA oligonucleotides, which function independently of transcription, was unaffected by ML-60218 treatment. In contrast, PTC readthrough mediated by DNA oligonucleotides was significantly reduced following ML-60218 treatment compared with the DMSO-treated control (Figures 4J and 4K). These findings indicate that suppressor tRNAs generated from DNA oligonucleotides are transcribed in an RNA Pol III-dependent manner.

Collectively, these results demonstrate that DNA oligonucleotides generate functional suppressor tRNAs that can be specifically inhibited by sequence-matched miRNAs and are transcribed in an RNA Pol III-dependent manner in cultured cells.

DISCUSSION

Here, we demonstrated that synthetic DNA oligonucleotides are capable of expressing functional suppressor tRNAs and inducing PTC readthrough in cultured cells. To our knowledge, this is the first report demonstrating that a short DNA fragment containing only a suppressor tRNA coding sequence and lacking additional RNA Pol III promoter elements can generate functional tRNAs that restore translation beyond a PTC. Given that suppressor tRNAs can be engineered to recognize any of the three termination codons and encode the desired amino acid, it is feasible that DNA-encoded suppressor tRNAs could be adapted to target a broader range of nonsense mutations. Our findings expand the repertoire of molecular therapy that can be used for PTC suppression.

Although the precise mechanism by which short single-stranded DNA oligonucleotides give rise to suppressor tRNAs remains to be elucidated, our findings provide several insights into this process. First, the reduction of DNA oligonucleotide-mediated PTC readthrough by RNA Pol III inhibition suggests that suppressor tRNAs are transcribed through an RNA Pol III-dependent mechanism. This is consistent with the presence of internal RNA Pol III promoter elements (A- and B-boxes) within tRNA-coding sequences, which can drive transcription independently of upstream promoter regions.[16] In addition, both sense and antisense DNA oligonucleotides were capable of inducing PTC readthrough, suggesting that either DNA strand may serve as a template for suppressor tRNA production. The molecular events that generate a transcriptionally competent template from short DNA oligonucleotides remain unclear. One possibility is that human DNA Pol θ extends single-stranded DNA through unimolecular stem-loop synthesis initiated by minimal 3′ microhomology,[17] potentially generating longer DNA templates suitable for suppressor tRNA transcription. Alternatively, the successful induction of PTC readthrough by the four-piece combination of split DNA oligonucleotides (Figure 2F) raises the possibility that these fragments assemble into a longer DNA intermediate through complementary annealing in cells, thereby forming a transcriptional template. These mechanisms are not mutually exclusive and warrant further investigation. Elucidating these processes will not only clarify the mechanism by which DNA oligonucleotides generate functional suppressor tRNAs but may also provide new insights into tRNA biogenesis and RNA Pol III-mediated transcription in mammalian cells.

The use of DNA oligonucleotides for suppressor tRNA expression has several potential advantages. DNA oligonucleotides are chemically stable and low cost and could provide longer-lasting effects than RNA oligonucleotides without the need for vector-based expression systems. In addition to these practical advantages, the potential for innate immune activation is an important consideration for nucleic acid-based therapeutics. Synthetic RNA molecules are well known to trigger innate immune sensors, including endosomal Toll-like receptors, such as TLR3, TLR7, and TLR8, as well as cytoplasmic RNA sensors such as RIG-I and MDA5.[18] However, the immunostimulatory properties of DNA oligonucleotides are highly dependent on their sequence composition, and unmethylated CpG motifs can induce TLR9-mediated immune activation.[19,20] Moreover, although DNA oligonucleotides may be less immunostimulatory than unmodified RNA, recent chemical modifications widely used in RNA therapeutics substantially reduce innate immune activation.[21] Therefore, careful sequence optimization together with appropriate chemical modifications of DNA oligonucleotides may be important for minimizing immune activation in future suppressor tRNA-based therapeutic applications. Consequently, DNA-based suppressor tRNA approaches may reduce the risk of unintended immune activation. However, the transient PTC readthrough efficiency observed with DNA oligonucleotides was lower than that mediated by plasmid- or RNA-based delivery, highlighting a current limitation of this approach. Future therapeutic development will require efficient and tissue-specific delivery of DNA oligonucleotides. Direct conjugation to targeting ligands, such as lipids, peptides, or antibodies, may represent a feasible strategy, analogous to delivery approaches developed for RNA therapeutics.[22] However, efficient intracellular delivery remains a major challenge. For example, only a small fraction of internalized oligonucleotides is thought to escape from endosomal compartments and reach the cytoplasm or nucleus, thereby limiting intracellular availability and therapeutic efficacy.[23,24] In addition, the limited stability of unmodified DNA oligonucleotides is likely to restrict their *in vivo* efficacy. Therefore, optimization of chemical modifications, together with advances in targeted delivery, endosomal escape, and efficient engagement of the transcriptional machinery, will likely be required to maximize the therapeutic potential of this approach. Another important consideration for future therapeutic development is the potential for off-target effects. These may arise from sequence-dependent interactions of DNA oligonucleotides with endogenous nucleic acids, as well as unintended suppressor tRNA-mediated readthrough of natural termination codons. Comprehensive transcriptomic and proteomic analyses will therefore be important to establish the specificity and safety profile of this approach.

For *in vivo* applications, several delivery technologies, including hydrodynamic injection and lipid nanoparticles, have already been established for nucleic acid therapeutics and could potentially be adapted for DNA oligonucleotides encoding suppressor tRNAs.[25,26]

Such approaches would facilitate evaluation in animal models of genetic diseases caused by nonsense mutations and help advance the therapeutic development of this strategy. Overall, this study establishes DNA oligonucleotides as a previously unrecognized format for generating suppressor tRNAs but also underscores the need to clarify the underlying transcriptional mechanism. Future work dissecting how tRNAs are produced from DNA oligonucleotides will not only improve their utility but may also reveal new modes of tRNA biogenesis and regulation in mammalian cells.

MATERIALS AND METHODS

Constructs and oligonucleotides

All recombinant DNA experiments were approved by the National Institute of Genetics (R3-19). To generate EGFP^{R73X}-expressing constructs, nonsense mutations were introduced at codon 73 (CGC, Arg) by site-directed mutagenesis (KLD reaction, NEB #M0554S) to replace the arginine codon with one of the three stop codons (TGA, TAA, or TAG), generating EGFP^{R73Xuga}, EGFP^{R73Xuaa}, and EGFP^{R73Xuag}, respectively. The following primer pairs were used for mutagenesis: EGFP^{R73Xuga}, forward 5′-GAtaccccgaccacatgaa gcagc-3′ and reverse 5′-Agctgaagcactgcacgccgtagg-3′; EGFP^{R73Xuaa}, forward 5′-agcTAAtaccccgaccacatgaagca-3′ and reverse 5′-gaag-cactgcacgccgtaggtca-3′; and EGFP^{R73Xuag}, forward 5′-agcTAGtaccc cgaccacatgaagca-3′ and reverse 5′-gaagcactgcacgccgtaggtca-3′. To generate the EGFP^{Q69X}-expressing construct, a nonsense mutation was introduced by site-directed mutagenesis, replacing the CAG (Gln, Q) codon at amino acid position 69 with a TAA (stop) codon using the following primers: 5′-atgcttcagccgctaccccgacca-3′ and 5′-gataccccgaccacatgaagcagc-3’. The plasmid contains hU6 promoter-derived tRNA followed by CAG promoter-derived mCherry in tandem was synthesized by GENEWIZ (currently AZENTA). Suppressor tRNA fragments were subcloned between EcoRI and BamHI restriction sites. DNA oligonucleotides and miRNAs were synthesized by FASMAC. RNA oligonucleotides were synthesized by GenScript Japan. Sequences of the DNA and RNA oligonucleotides used in this study are provided in Table S1.

Design of the oligonucleotides

The tRT6 suppressor tRNA sequence was selected based on the previously reported construct described by Albers et al.[9] The sequences of sup-tRNA-Yuag and sup-tRNA-Quaa were designed based on entries in the GtRNAdb database. Specifically, sup-tRNA-Yuag was derived from human tRNA-Tyr-GTA-2 lacking an intron,[8] whereas sup-tRNA-Quaa was derived from human tRNA-Gln-TTG-3. In both constructs, the anticodon was modified to recognize the corresponding stop codon, and an additional 3′ CCA sequence was appended to the designed DNA sequence.

Cell culture and transfection

HEK293T cells were cultured in Dulbecco’s modified Eagle’s medium high glucose (Gibco, #11965092) supplemented with 10% fetal bovine serum (Nichirei Biosciences, #175012) and 1% penicillin-streptomycin (Gibco, #15070063). Cells were passaged on Poly-D-Lysine (Gibco, #A3890401) courted cover glass (Matsunami Glass, #83–0177) in 24-well plate. 500 ng of plasmids, 100 pmole of oligonucleotides, or 100 pmole of miRNAs were transfected into HEK293T cells by lipofectamine 3000 (Invitrogen, #L3000008) following the manufacture protocol.

Pol III inhibitor treatment

The Pol III inhibitor ML-60218 (MedChemExpress, HY-122122) was used at a final concentration of 25 μM.[27] HEK293T cells were first transfected with the EGFP^{R73Xuga} reporter plasmid. Twenty-four hours later, 500 ng of plasmid DNA or 100 pmol of DNA oligonucleotides were transfected into the cells. Six hours after the second transfection, the culture medium was replaced with fresh medium containing 25 μM ML-60218 or an equivalent volume of DMSO (control). Cells were fixed 48 h after the second transfection and subjected to fluorescence imaging.

Image analysis

Cells were fixed 4% paraformaldehyde in phosphate buffered saline (PBS) for 15 min at room temperature and then washed three times with PBS. Nuclei were stained with DAPI (Dojindo #D523; final concentration 1 μg/mL in PBS) for at least 30 min at room temperature. Cover glasses ware mounted on slide glass by ProLong Gold Antifade Mountant (Thermo Fisher Scientific, #P36934). Fluorescent imaging was performed on EVOS M5000 Imaging System (Thermo Fisher Scientific) and Olympus confocal microscope FV3000 (Evident) with Olympus UPlanXApo 10X/0.40 NA objective lens.

Northern blotting

Northern blotting was performed as previously described with modifications for fluorescent probe detection.[28,29] Briefly, total RNA was extracted from HEK293T cells using TRIzol Reagent (Thermo Fisher Scientific, #15596026), and small RNAs were subsequently purified using the Zymo RNA Clean & Concentrator kit (Zymo Research, #R1016). 100 ng of small RNAs were resolved by electrophoresis on a Novex TBE-Urea 10% gel (Thermo Fisher Scientific, #EC68755BOX), transferred onto Hybond N+ membranes (Cytiva, #RPN1510B) at 12 V for 30 min using the PoweredBLOT 2 M system (ATTO, #WSE-4125), UV crosslinked, and hybridized overnight at 42°C with Cy5-labeled DNA probes (300 pmol per membrane). Cy5-labeled probes targeting tRT6 suppressor tRNA and DY782-labeled probes targeting human U6 snRNA[30] were used for detection, with U6 snRNA serving as a loading control. Cy5 fluorescence signals were directly detected using a ChemiDoc Touch Imaging System (Bio-Rad). All DNA probes were synthesized by Eurofins Genomics. The sequences of the DNA probes used in this study are provided in Table S1.

tRNA library preparation for qPCR

For tRNA library preparation, 10 μg of total RNA was treated with TURBO DNase I (Thermo Fisher Scientific, #AM2238) at 37°C for 30 min to remove contaminating genomic DNA. The RNA was subsequently deacylated to remove amino acids from tRNAs, followed by isolation of the small RNA fraction using the Zymo RNA Clean & Concentrator kit (Zymo Research, #R1016), according to the Nano-tRNAseq protocol.[15] Briefly, 5′ and 3′ splint adapters were ligated to

purified small RNAs, followed by ligation of RTA adapters. Reverse transcription was then performed using Maxima H Minus Reverse Transcriptase (Thermo Fisher Scientific, #EP0752) at 60°C for 1 h, and the resulting tRNA libraries were analyzed by qPCR using TB Green Premix Ex Taq II (Takara Bio, #RR820A) on a CFX96 Touch Real-Time PCR Detection System (Bio-Rad).

Quantification and statistical analysis

Fluorescence signal densities and intensities were quantified in Fiji (ImageJ) using available plugins and MATLAB 2024a (MathWorks). Regions of interest (ROIs) corresponding to transfected cells were generated using the Cell Magic Wand Tool (https://github.com/fitzlab/CellMagicWand). For GFP-positive cell quantification, the percentage of GFP-positive cells among total DAPI-positive nuclei was determined. The GFP fluorescence threshold was manually defined based on background fluorescence and uniformly applied to all images. DAPI-positive nuclei and GFP-positive cells were segmented using the Watershed algorithm to separate adjacent objects, and particles were counted using the Analyze Particles function in Fiji (ImageJ). The same analysis parameters were applied to all GFP images to minimize observer bias and ensure objective quantification. Data are presented as mean with standard deviation. Statistical analyses and making graphs were performed using GraphPad Prism 10 (https://www.graphpad.com/). Single comparisons were performed using Student's *t* test and multiple comparisons were performed using one-way analysis of variance (ANOVA) with post hoc Tukey's tests or Dunnett's test. Asterisks indicate significance of values as follows $*p < 0.05$, $**p < 0.01$.

DATA AND CODE AVAILABILITY

The data underlying this article are available in the article and in its supplemental information.

ACKNOWLEDGMENTS

We thank Drs. K. Saito and K. Miyoshi (National Institute of Genetics) for providing the Northern blotting protocol and for helpful discussions. This work was supported by grants from NIG postdoctoral fellow to H.Y.; and ROIS Strategic Research Projects (2025-SRP-22) to K.Y.

AUTHOR CONTRIBUTIONS

K.Y. and P.Z. conceived the project. K.Y. supervised the project. H.Y., K.Y., and P.Z. designed the experiments. H.Y. performed all experiments and analyzed the data. H.Y. and K.Y. interpreted the data and wrote the manuscript.

DECLARATION OF INTERESTS

P.Z. is an employee of Verve Therapeutics, United States, and an affiliate member of the Broad Institute, Cambridge, United States.

DECLARATION OF GENERATIVE AI AND AI-ASSISTED TECHNOLOGIES IN THE MANUSCRIPT PREPARATION PROCESS

During the preparation of this work, we used ChatGPT (GPT-5) in order to refine sentence structure. After using this tool, we reviewed and edited the content as needed and take full responsibility for the content of the publication.

SUPPLEMENTAL INFORMATION

Supplementary data related to this article can be found online at https://doi.org/10.1016/j.omtn.2026.103110

REFERENCES

1. Mort, M., Ivanov, D., Cooper, D.N., and Chuzhanova, N.A. (2008). A meta-analysis of nonsense mutations causing human genetic disease. Hum. Mutat. *29*, 1037–1047. https://doi.org/10.1002/humu.20763.
2. Wangen, J.R., and Green, R. (2020). Stop codon context influences genome-wide stimulation of termination codon readthrough by aminoglycosides. eLife *9*, e52611. https://doi.org/10.7554/eLife.52611.
3. Welch, E.M., Barton, E.R., Zhuo, J., Tomizawa, Y., Friesen, W.J., Trifillis, P., Paushkin, S., Patel, M., Trotta, C.R., Hwang, S., et al. (2007). PTC124 targets genetic disorders caused by nonsense mutations. Nature *447*, 87–91. https://doi.org/10.1038/nature05756.
4. Gurzeler, L.A., Link, M., Ibig, Y., Schmidt, I., Galuba, O., Schoenbett, J., Gasser-Didierlaurant, C., Parker, C.N., Mao, X., Bitsch, F., et al. (2023). Drug-induced eRF1 degradation promotes readthrough and reveals a new branch of ribosome quality control. Cell Rep. *42*, 113056. https://doi.org/10.1016/j.celrep.2023.113056.
5. Dabrowski, M., Bukowy-Bieryllo, Z., and Zietkiewicz, E. (2018). Advances in therapeutic use of a drug-stimulated translational readthrough of premature termination codons. Mol. Med. *24*, 25. https://doi.org/10.1186/s10020-018-0024-7.
6. Temple, G.F., Dozy, A.M., Roy, K.L., and Kan, Y.W. (1982). Construction of a functional human suppressor tRNA gene: an approach to gene therapy for beta-thalassaemia. Nature *296*, 537–540. https://doi.org/10.1038/296537a0.
7. Lueck, J.D., Yoon, J.S., Perales-Puchalt, A., Mackey, A.L., Infield, D.T., Behlke, M.A., Pope, M.R., Weiner, D.B., Skach, W.R., McCray, P.B., and Ahern, C.A. (2019). Engineered transfer RNAs for suppression of premature termination codons. Nat. Commun. *10*, 822. https://doi.org/10.1038/s41467-019-08329-4.
8. Wang, J., Zhang, Y., Mendonca, C.A., Yukselen, O., Muneeruddin, K., Ren, L., Liang, J., Zhou, C., Xie, J., Li, J., et al. (2022). AAV-delivered suppressor tRNA overcomes a nonsense mutation in mice. Nature *604*, 343–348. https://doi.org/10.1038/s41586-022-04533-3.
9. Albers, S., Allen, E.C., Bharti, N., Davyt, M., Joshi, D., Perez-Garcia, C.G., Santos, L., Mukthavaram, R., Delgado-Toscano, M.A., Molina, B., et al. (2023). Engineered tRNAs suppress nonsense mutations in cells and in vivo. Nature *618*, 842–848. https://doi.org/10.1038/s41586-023-06133-1.
10. Colella, P., Ronzitti, G., and Mingozzi, F. (2018). Emerging Issues in AAV-Mediated. Mol. Ther. Methods Clin. Dev. *8*, 87–104. https://doi.org/10.1016/j.omtm.2017.11.007.
11. Hou, X., Zaks, T., Langer, R., and Dong, Y. (2021). Lipid nanoparticles for mRNA delivery. Nat. Rev. Mater. *6*, 1078–1094. https://doi.org/10.1038/s41578-021-00358-0.
12. Bordeira-Carriço, R., Ferreira, D., Mateus, D.D., Pinheiro, H., Pêgo, A.P., Santos, M.A.S., and Oliveira, C. (2014). Rescue of wild-type E-cadherin expression from nonsense-mutated cancer cells by a suppressor-tRNA. Eur. J. Hum. Genet. *22*, 1085–1092. https://doi.org/10.1038/ejhg.2013.292.
13. Specht, C., Tapia, A., Penrod, S., Soriano, G.A., Awawdeh, A., Alshawi, S.A., White, C.A., Beaudoin, J.D., Doud, E.H., Vargas-Rodriguez, O., et al. (2025). An engineered glutamic acid tRNA for efficient suppression of pathogenic nonsense mutations. Nucleic Acids Res. *53*, gkaf532. https://doi.org/10.1093/nar/gkaf532.
14. Cui, Z., Wu, Y., Mureev, S., and Alexandrov, K. (2018). Oligonucleotide-mediated tRNA sequestration enables one-pot sense codon reassignment in vitro. Nucleic Acids Res. *46*, 6387–6400. https://doi.org/10.1093/nar/gky365.
15. Lucas, M.C., Pryszcz, L.P., Medina, R., Milenkovic, I., Camacho, N., Marchand, V., Motorin, Y., Ribas de Pouplana, L., and Novoa, E.M. (2024). Quantitative analysis of tRNA abundance and modifications by nanopore RNA sequencing. Nat. Biotechnol. *42*, 72–86. https://doi.org/10.1038/s41587-023-01743-6.
16. Arimbasseri, A.G., and Maraia, R.J. (2016). RNA Polymerase III Advances: Structural and tRNA Functional Views. Trends Biochem. Sci. *41*, 546–559. https://doi.org/10.1016/j.tibs.2016.03.003.
17. Carvajal-Maldonado, D., Li, Y., Returan, M., Averill, A.M., Doublié, S., and Wood, R.D. (2024). Dynamic stem-loop extension by Pol θ and templated insertion during DNA repair. J. Biol. Chem. *300*, 107461. https://doi.org/10.1016/j.jbc.2024.107461.

18. Liu, G., and Gack, M.U. (2020). Distinct and Orchestrated Functions of RNA Sensors in Innate Immunity. Immunity *53*, 26–42. https://doi.org/10.1016/j.immuni.2020.03.017.

19. Bauer, S., Pigisch, S., Hangel, D., Kaufmann, A., and Hamm, S. (2008). Recognition of nucleic acid and nucleic acid analogs by Toll-like receptors 7, 8 and 9. Immunobiology *213*, 315–328. https://doi.org/10.1016/j.imbio.2007.10.010.

20. Zhao, H., Hemmi, H., Akira, S., Cheng, S.H., Scheule, R.K., and Yew, N.S. (2004). Contribution of Toll-like receptor 9 signaling to the acute inflammatory response to nonviral vectors. Mol. Ther. *9*, 241–248. https://doi.org/10.1016/j.ymthe.2003.11.012.

21. Neill, B., Romero, A.R., and Fenton, O.S. (2024). Advances in Nonviral mRNA Delivery Materials and Their Application as Vaccines for Melanoma Therapy. ACS Appl. Bio Mater. *7*, 4894–4913. https://doi.org/10.1021/acsabm.3c00721.

22. Juliano, R.L. (2016). The delivery of therapeutic oligonucleotides. Nucleic Acids Res. *44*, 6518–6548. https://doi.org/10.1093/nar/gkw236.

23. Sahay, G., Querbes, W., Alabi, C., Eltoukhy, A., Sarkar, S., Zurenko, C., Karagiannis, E., Love, K., Chen, D., Zoncu, R., et al. (2013). Efficiency of siRNA delivery by lipid nanoparticles is limited by endocytic recycling. Nat. Biotechnol. *31*, 653–658. https://doi.org/10.1038/nbt.2614.

24. Roberts, T.C., Langer, R., and Wood, M.J.A. (2020). Advances in oligonucleotide drug delivery. Nat. Rev. Drug Discov. *19*, 673–694. https://doi.org/10.1038/s41573-020-0075-7.

25. Sufian, M.A., and Ilies, M.A. (2023). Lipid-based nucleic acid therapeutics with in vivo efficacy. Wiley Interdiscip. Rev. Nanomed. Nanobiotechnol. *15*, e1856. https://doi.org/10.1002/wnan.1856.

26. Suda, T., Yokoo, T., Kanefuji, T., Kamimura, K., Zhang, G., and Liu, D. (2023). Hydrodynamic Delivery: Characteristics, Applications, and Technological Advances. Pharmaceutics *15*, 1111. https://doi.org/10.3390/pharmaceutics15041111.

27. Miyoshi, K., Otsubo, K., Kaneko, S., Terauchi, M., Hatoyama, Y., Noguchi, H., Kanemaki, M.T., and Saito, K. (2025). The mammalian rapid tRNA decay pathway is critical for N^7-methylguanosine-hypomodified tRNA degradation under physiological conditions. Preprint at bioRxiv. https://doi.org/10.1101/2025.11.05.686800.

28. Kaneko, S., Miyoshi, K., Tomuro, K., Terauchi, M., Tanaka, R., Kondo, S., Tani, N., Ishiguro, K.I., Toyoda, A., Kamikouchi, A., et al. (2024). Mettl1-dependent m. Nat. Commun. *15*, 8147. https://doi.org/10.1038/s41467-024-52389-0.

29. Otsubo, K., Nagura, M., Terauchi, M., Noguchi, H., Kanemaki, M.T., Miyoshi, K., and Saito, K. (2026). m^1A58 governs two steps of human initiator-methionine tRNA metabolism: maturation and XRN2-mediated decay. Preprint at bioRxiv. https://doi.org/10.64898/2026.05.28.728624.

30. Ma, J., Han, H., Huang, Y., Yang, C., Zheng, S., Cai, T., Bi, J., Huang, X., Liu, R., Huang, L., et al. (2021). METTL1/WDR4-mediated m. Mol. Ther. *29*, 3422–3435. https://doi.org/10.1016/j.ymthe.2021.08.005.

OMTN, Volume *37*

Supplemental information

DNA oligonucleotides encoding suppressor tRNA induce readthrough of premature termination codon

Haruka Yamamoto, Peixin Zhu, and Keisuke Yonehara

Table S1. Sequences of DNA and RNA oligonucleotides

Name	Sequence (5'-3')	
tRT6-sense	GGCTCTGTGGCGCAATGGATAGCGCATTGGACTTCAAATTCAAAGGTTGCGGGTTCGAGTCCCGTCAGAGTCGCCA	DNA
tRT6-antisense	TGGCGACTCTGACGGGACTCGAACCCGCAACCTTTGAATTTGAAGTCCAATGCGCTATCCATTGCGCCACAGAGCC	DNA
tRT6-mut	GGCTCTGTGGCGCAATGGATAGCGCATTGGACTTCGAATTCAAAGGTTGCGGGTTCGAGTCCCGTCAGAGTCGCCA	DNA
suptRNA-Quaa	GGCCCCATGGTGTAATGGTtAGCACTCTGGACTTTAAATCCAGCGATCCGAGTTCAAATCTCGGTGGGACCTCCA	DNA
suptRNA-Yuag	CCTTCGATAGCTCAGTTGGTAGAGCGGAGGACTCTAGATCCTTAGGTCGCTGGTTCGATTCCGGCTCGAAGGACCA	DNA
Oligo 1	GGCTCTGTGGCGCAATGGATAGCGCATTGGACTTCAAA	DNA
Oligo 2	AAGTCCAATGCGCTATCCATTGCGCCACAGAGCC	DNA
Oligo 3	TTCAAAGGTTGCGGGTTCGAGTCCCGTCAGAGTCGCCA	DNA
Oligo 4	TGGCGACTCTGACGGGACTCGAACCCGCAACCTTTGAATTTG	DNA
tRT6-RNA	GGCUCUGUGGCGCAAUGGAUAGCGCAUUGGACUUCAAAUUCAAAGGUUGCGGGUUCGAGUCCCGUCAGAGUCGCCA	RNA
tRT6-mut-RNA	GGCUCUGUGGCGCAAUGGAUAGCGCAUUGGACUUCGAAUUCAAAGGUUGCGGGUUCGAGUCCCGUCAGAGUCGCCA	RNA
tRT6 miRNA	CGAACCCGCAACCUUUGAAUUUGA	RNA
Control miRNA	UUGAACUCGGAUCGCUGGAUUUAA	RNA
tRT6-Cy5	CCCGCAACCTTTGAATTTGA	DNA
human-U6-snRNA-DY782	TGGAACGCTTCACGAATTTG	DNA
tRT6-qPCR-F	CCCGCAACCTTTGAATTTGA	DNA
tRNA-Ala-AGC-2-1-qPCR-F	TAGCTCAGTGGTAGAGCGCG	DNA
tRNA-qPCR-common-R	CTAAGAGCAAGAAGAAGCCT	DNA
